# Tuberculosis burden in Saudi Arabia: retrospective analysis of national surveillance data, 2012–2024, with future forecasts

**DOI:** 10.1186/s12879-026-14222-5

**Published:** 2026-08-17

**Authors:** Intisar Alruwaili, Shandana Riaz, Hasan Ejaz, Muharib Alruwaili, Fahda Alsharief, Bayan Fallatah, Lienda Bashier Eltayeb, Bi Bi Zainab Mazhari, Muhammad Umer Khan

**Affiliations:** 1 Department of Clinical Laboratory Sciences, College of Applied Medical Sciences, Jouf University, Sakaka, 72388 Saudi Arabia; 2 Institute of Molecular Biology and Biotechnology, The University of Lahore, Lahore, Pakistan; 3 Department of Medical Laboratory Sciences, College of Applied Medical Sciences, Prince Sattam Bin AbdulAziz University- Al-Kharj, Riyadh, 11942 Saudi Arabia; 4 Department of Clinical Laboratory Sciences, College of Applied Medical Sciences, Jouf University, Qurayyat, 75911 Saudi Arabia

**Keywords:** Tuberculosis, Saudi Arabia, Spatiotemporal analysis, Segmented regression, ARIMA forecasting, Epidemiology

## Abstract

Saudi Arabia faces a major tuberculosis (TB) challenge, potentially amplified by a large expatriate population from endemic areas, yet long-term national analyses remain limited. A retrospective analysis of Ministry of Health surveillance data was conducted for 2012–2024. Temporal trends were evaluated using segmented regression, while spatial heterogeneity across 20 health regions was assessed using burden and incidence analyses; distributions by sex, nationality, age, and TB type were examined. Future TB incidence rates were projected to 2029 using ARIMA and exponential smoothing (ETS) models; given the small number of annual observations, these projections are exploratory. A total of 37,477 TB cases were reported. A 2013 change point was followed by a decline of about 70 cases per year to 2024. Incidence fell during the 2020 pandemic and is currently around 7–8 cases per 100,000 population, nationally. Burden was concentrated in Jeddah and Riyadh, while incidence analyses additionally identified Jazan’s elevated population-adjusted notification rate. Cases were more frequent among males (67.5–79.7%) and adults aged ≥ 15 years (~ 97% of notifications with age data); non-Saudi residents accounted for 42.0-57.5%, exceeding 50% in several years. Pulmonary TB comprised 73.6–79.0% of cases. Exploratory forecasts projected broadly stable incidence to 2029, with wide prediction intervals. Although TB burden has declined since 2013, national incidence has shown little further reduction after 2020. Exploratory forecasts suggest broadly stable incidence through 2029, although considerable uncertainty remains. Strengthened surveillance, targeted screening, and region-specific interventions are needed to advance WHO End TB targets.

**Supplementary Information:**

The online version contains supplementary material available at https://doi.org/10.1186/s12879-026-14222-5.

## Clinical trial number

Not applicable.

## Introduction

Tuberculosis (TB), which is induced by Mycobacterium tuberculosis, is one of the important infectious illnesses that continue to have a significant effect on morbidity and mortality around the globe [1–3]. As per the world health organization (WHO) TB Report 2024, about 10.8 million people became infected with TB, while 1.25 million lost their lives due to TB infection during 2023, showing that the condition remains prevalent globally [4, 5]. However, despite several attempts to control the disease through Directly Observed Treatment Short course (DOTS), along with rapid diagnostics such as molecular testing, the level of success is still inadequate. This does not meet the WHO’s End TB Strategy goal of achieving a 90% reduction in tuberculosis incidence by 2030 [4, 6]. The incidence rate of tuberculosis continues to be excessively high in developing and underdeveloped countries. It is also a significant public health issue in developed countries, where the burden of disease is disproportionately concentrated among migrant communities because of socioeconomic and healthcare-access disparities [7, 8].

The epidemiological landscape of Saudi Arabia makes it a unique case study for TB. Even though Saudi Arabia is classified as a developed nation, it has a considerable number of immigrants, which is more than 13 million, who hail from TB-endemic countries like South and Southeast Asia and sub-Saharan Africa. It is pertinent to note that this factor significantly contributes to the epidemiology of TB in Saudi Arabia [9, 10]. Furthermore, Saudi Arabia serves as an important hub for pilgrimage and tourism, making the epidemiology of TB more complicated in the country. The interplay of imported cases with constant human mobility affects local disease transmission [10, 11]. Concurrently, the country has been able to make significant contributions towards TB control programs. This includes mandatory screening among applicants for labor permits, a nationally coordinated surveillance program administered by the Ministry of Health (MOH), and ensuring access to appropriate treatment regimens according to the WHO guidelines [4, 6].

Even with all these initiatives, systematic analysis on the national level regarding the long-term trends of TB in Saudi Arabia is relatively rare in the scientific literature. In most cases, only specific regions or institutions are analyzed, while other researches cover specific populations or use a short time interval [12, 13]. Although annual Ministry of Health surveillance reports provide valuable epidemiological information, they do not offer integrated analyses of long-term temporal trends, population-adjusted regional incidence patterns, demographic heterogeneity, or future disease projections. Consequently, a comprehensive national assessment incorporating spatiotemporal epidemiology and forecasting remains lacking (https://www.moh.gov.sa/en/ministry/statistics/book/pages/default.aspx) [14]. The importance of this analytical limitation is increasingly becoming apparent amid the coronavirus pandemic. The coronavirus pandemic caused major disruption to TB care globally. These disruptions involved fewer diagnoses, shifts in health-seeking behavior, and reallocation of healthcare facilities to pandemic responses [15].

Segmented (piecewise linear) regression is a standard statistical method for identifying changes in the direction or slope of epidemiological trends over time. Similar approaches are commonly implemented in joinpoint regression analyses. This tool is widely employed in TB surveillance as a way of identifying critical points where there is likely to be a change in program efficiency or in population risk [16–18]. Simultaneously, autoregressive integrated moving average (ARIMA) models represent common forecasting techniques for infectious disease outbreaks. These provide a probabilistic forecast that can facilitate the planning of resource deployment and decision-making [7, 19]. Incorporating these techniques into a nationwide longitudinal study, accounting for regional differences, strains of TB, sub-population differences, and projections for the future, would offer an even more robust evidence base compared to surveillance reports alone.

This study addresses the existing knowledge gap in national TB surveillance by providing a comprehensive spatiotemporal and forecasting analysis of TB in Saudi Arabia. The analysis specifically evaluates temporal trends, demographic patterns, regional burden, population-adjusted incidence rates across the 13 administrative regions, and future projections of TB incidence. Segmented regression was used to examine shifts in TB trends over time, while ARIMA models were used to forecast TB incidence rates for the period 2025–2029. This research contributes a holistic evidentiary foundation through its incorporation of spatiotemporal analysis and predictive modeling to aid public health decisions in enhancing TB control efforts in line with the WHO End TB Strategy objectives.

## Materials and methods

### Study design and data sources

A retrospective longitudinal study was conducted using national tuberculosis (TB) surveillance data reported by the Ministry of Health (MOH), Saudi Arabia, for the period 2012–2024. Annual TB notification data were extracted from the official MOH Statistical Yearbooks and Health Statistics reports published between 2012 and 2024. These reports are publicly available through the MOH Statistical Yearbook portal: (https://www.moh.gov.sa/en/Ministry/Statistics/book/Pages/default.aspx) [14]. Data were obtained from the annual communicable disease and tuberculosis surveillance tables contained within each report. Extracted variables included total TB notifications, pulmonary tuberculosis (PTB), extrapulmonary tuberculosis (EPTB), sex, nationality (Saudi and non-Saudi), age-group distributions, and regional case counts across the 20 MOH health regions. For the population-adjusted (incidence) analysis, regional case counts were mapped to the 13 administrative regions of Saudi Arabia, for which official annual resident-population estimates are published. Annual population denominators were obtained from the General Authority for Statistics (GASTAT), which produces population estimates for the 13 administrative regions (https://www.stats.gov.sa/en/), together with the corresponding MOH statistical reports.

All data were manually extracted from the original PDF reports and excel files, and compiled into a standardized database. To ensure data accuracy and consistency, extracted values were independently cross-checked against the original MOH source documents by three investigators prior to statistical analysis. Any discrepancies identified during verification were resolved through consensus review of the original reports. All source documents were accessed between January and March 2025.

The database consisted exclusively of aggregated, anonymized, publicly available surveillance records. Therefore, ethical approval and informed consent were not required in accordance with institutional and national research guidelines [13].

### Study area and health region classification

**Fig. 1:**
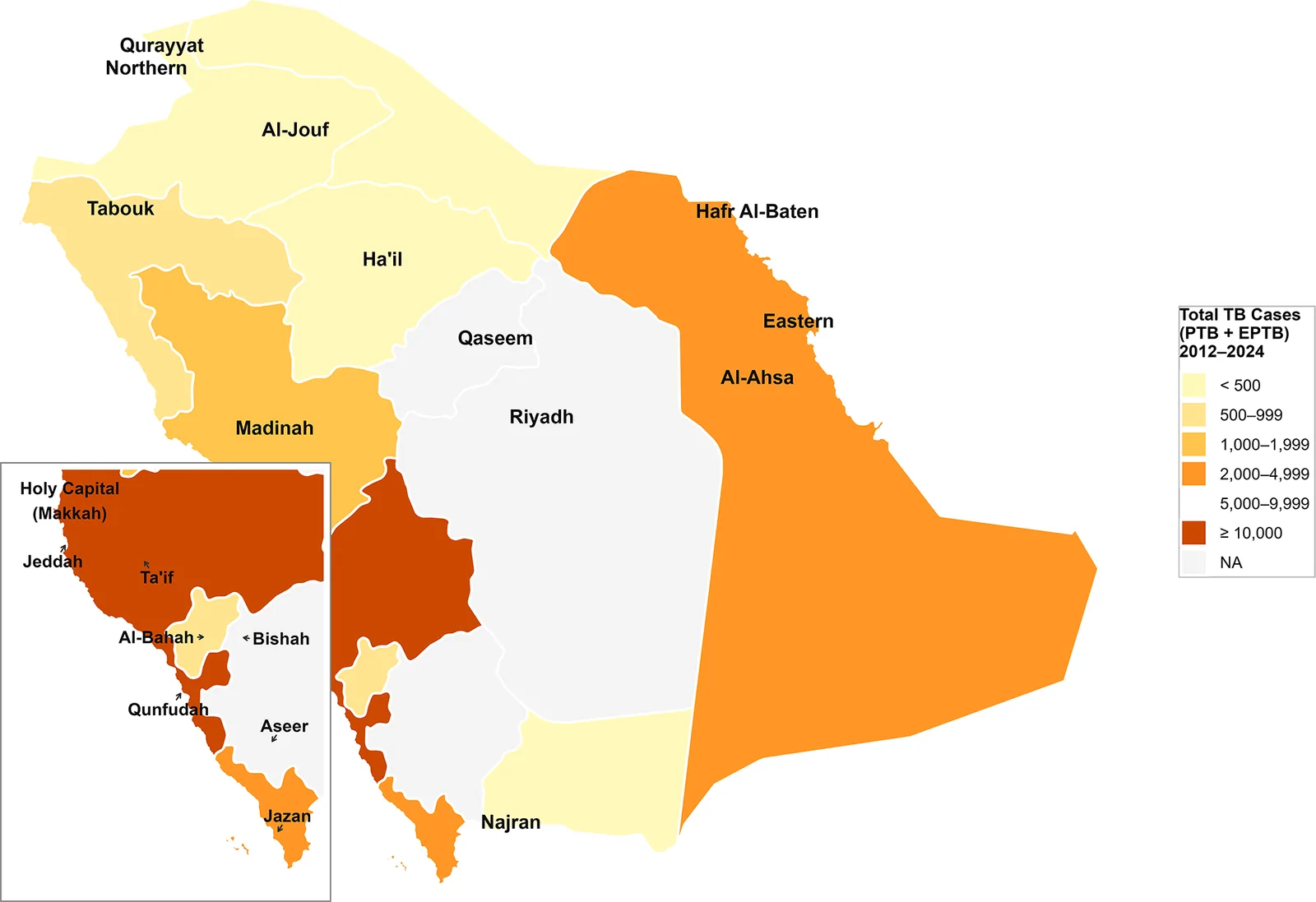
Ministry of Health (MOH) health regions of Saudi Arabia. Map representing the 20 health regions defined by MOH in relation to tuberculosis (PTB + EPTB) surveillance and analysis between 2012 to 2024

Saudi Arabia is officially divided into 13 administrative regions, whereas the Ministry of Health (MOH) conducts disease surveillance and TB notification through 20 health (reporting) regions corresponding to major cities and administrative areas. Absolute case counts and the cumulative burden analysis were reported using these 20 regions, comparable across the study period [20]. For the population-adjusted (incidence) analysis, case counts were aggregated to the 13 administrative regions, since official resident-population denominators are published at that level; a crosswalk between the two systems is provided in Supplementary Table S3. The spatial layout of the MOH health regions is shown in Fig. 1, which serves as the geographic basis for the regional analyses in this study.

### Case definition and classification

The cases of TB were categorized using the surveillance case definitions set out by the MOH of Saudi Arabia. Pulmonary tuberculosis (PTB) was described as the involvement of the lung parenchyma by TB, which could be confirmed either by laboratory testing or clinically. Extra-pulmonary tuberculosis (EPTB) was considered to be TB occurring in sites other than the lungs, such as the lymph nodes, pleura, bones/joints, genitourinary system, and central nervous system [21]. These cases were classified into PTB and EPTB, depending on the main site represented by the infection. Notifications of TB cases were provided for the total number of individuals annually, categorized according to TB types. Demographic data available were sex (male/female), nationality (Saudi/non-Saudi), and age categories (< 5, 5–14, 15–24, 25–34, 35–44, 45–54, 55–64, and ≥ 65 years). The non-Saudi group refers to people who are not Saudi nationals and includes expatriates and tourists because the surveillance system does not classify these two different groups.

### Descriptive and temporal analyses

Country-level TB burden and incidence were analyzed annually from 2012 to 2024. The annual proportions of PTB and EPTB were computed, along with their distribution by sex, nationality, and age group. To assess temporal trends and identify any change in their direction, segmented regression was applied to the annual number of TB cases using the segmented package in R [22, 23]. A single-breakpoint model was fitted, with the breakpoint position determined from the model fit; the slopes before and after the breakpoint were then compared to characterize how TB cases changed over time.

### Spatial and regional analysis

The spatial variability of TB burden was explored among the 20 health regions of the MOH based on the annual aggregate number of cases for the period 2012–2024. The regional numbers of PTB, EPTB, and TB cases were arranged in matrix form by region and year for spatiotemporal analysis. Heat maps were constructed to present the geographical and temporal distribution of TB in the regions. Cumulative numbers of cases were also determined for all regions to establish which regions had sustained a TB burden. In order to delve deeper into the regional trend, the top ten regions that had the greatest cumulative burden of TB were selected, and time series plots for PTB and EPTB were created separately. Because official annual resident-population figures are published only for the 13 administrative regions of Saudi Arabia, and not for the 20 MOH reporting regions, case counts were mapped to the 13 administrative regions using a defined geographic crosswalk. Population-adjusted notification rates per 100,000 population were then calculated at the administrative-region level for pulmonary TB (PTB), extrapulmonary TB (EPTB), and total TB, using the annual regional population published by the General Authority for Statistics (GASTAT) as the denominator. As these denominators were available through 2022, the population-adjusted regional analysis covers 2012–2022, whereas the national temporal and forecasting analyses cover 2012–2024. For each administrative region, the annual case numerator, population denominator, calculated rate, and source, together with the crosswalk from the 20 MOH reporting regions, are provided in Supplementary Table S3.

### Demographic subgroup analysis

TB demographics were analyzed by stratifying surveillance data according to sex, nationality, and age group. Annual TB notifications among males and females were compared to evaluate sex-specific temporal patterns. Nationality-based stratification was performed to assess differences in annual TB notifications between Saudi and non-Saudi individuals. For age-group analyses, cases were categorized into eight predefined age groups to examine temporal patterns of pulmonary tuberculosis (PTB) and extra pulmonary tuberculosis (EPTB) across the lifespan. For all demographic variables, both absolute case counts and proportional distributions were calculated to provide a comprehensive characterization of the represented populations.

### Time-series forecasting

National TB incidence rates were forecasted separately for pulmonary tuberculosis (PTB), extrapulmonary tuberculosis (EPTB), and total TB using autoregressive integrated moving average (ARIMA) models fitted to annual incidence rate data from 2012 to 2024 [24]. Before the model fitting, stationarity was assessed using the Augmented Dickey-Fuller (ADF) and Kwiatkowski-Phillips-Schmidt-Shin (KPSS) tests. Based on the stationarity assessment, first-order differencing was applied where appropriate to achieve stationarity and facilitate model identification [25]. To account for potential disruptions in TB surveillance and healthcare utilization during the COVID-19 pandemic, a binary intervention variable was incorporated into the models, coded as 1 for 2020 and 0 otherwise. Model orders were selected by minimising the small-sample corrected Akaike Information Criterion (AICc) across candidate ARIMA specifications, implemented in Python (statsmodels) [23].

Model adequacy was evaluated through residual diagnostics, including visual inspection of residual plots, autocorrelation functions, and the Ljung-Box test for residual autocorrelation. As a complementary forecasting approach, exponential smoothing state-space models with additive damped trends [ETS(A, Ad, N)] were also fitted to each series to assess the consistency of projected trends across alternative forecasting frameworks.

Forecasts were generated for the period 2025–2029 on the incidence rate scale, and uncertainty was quantified using 80% and 95% prediction intervals. Selected model orders, intervention coefficients, AICc values, stationarity and residual diagnostic test results, ETS specifications, and annual forecasts with 80% and 95% prediction intervals are reported in Supplementary Table S2. Given the limited number of annual observations, forecast results were interpreted cautiously, with particular consideration of the uncertainty reflected in the prediction intervals.

### Statistical software

Data management and preliminary tabulation were done using Microsoft Excel. Segmented regression analysis and spatial mapping were computed using R software version 4.5.2 (R Foundation for Statistical Computing, Vienna, Austria), while ARIMA and ETS time-series forecasting was performed in Python version 3.12 using the statsmodels library. All analyses were run in the RStudio integrated development environment (version 2026.01.0 + 392; Posit Software). Data preparation and visualization were accomplished using standard R libraries like *readxl*, *dplyr*, *tidyr*, *ggplot2*, *scales*, *patchwork*, *RColorBrewer*, *viridis*, *ggrepel*, *stringr*, *forcats*, and *Cairo*, respectively. *Time-series forecasting was performed in Python using the statsmodels library*,* and segmented regression in R using the segmented package*.

## Results

### National TB trends in Saudi Arabia (2012–2024)

From 2012 to 2024, the total number of TB cases diagnosed in Saudi Arabia was 37,477. Table 1 demonstrates that there has been a rise and fall in the number of cases over the years, reaching its lowest figure of 2,508 cases in 2012 and its highest of 3,364 cases in 2018. A comparable peak was recorded in 2015 (3,346 cases), followed by a sharp drop in 2020 (2,511 cases) amid the onset of the COVID-19 pandemic and its impact on TB diagnosis and reporting.

**Fig. 2:**
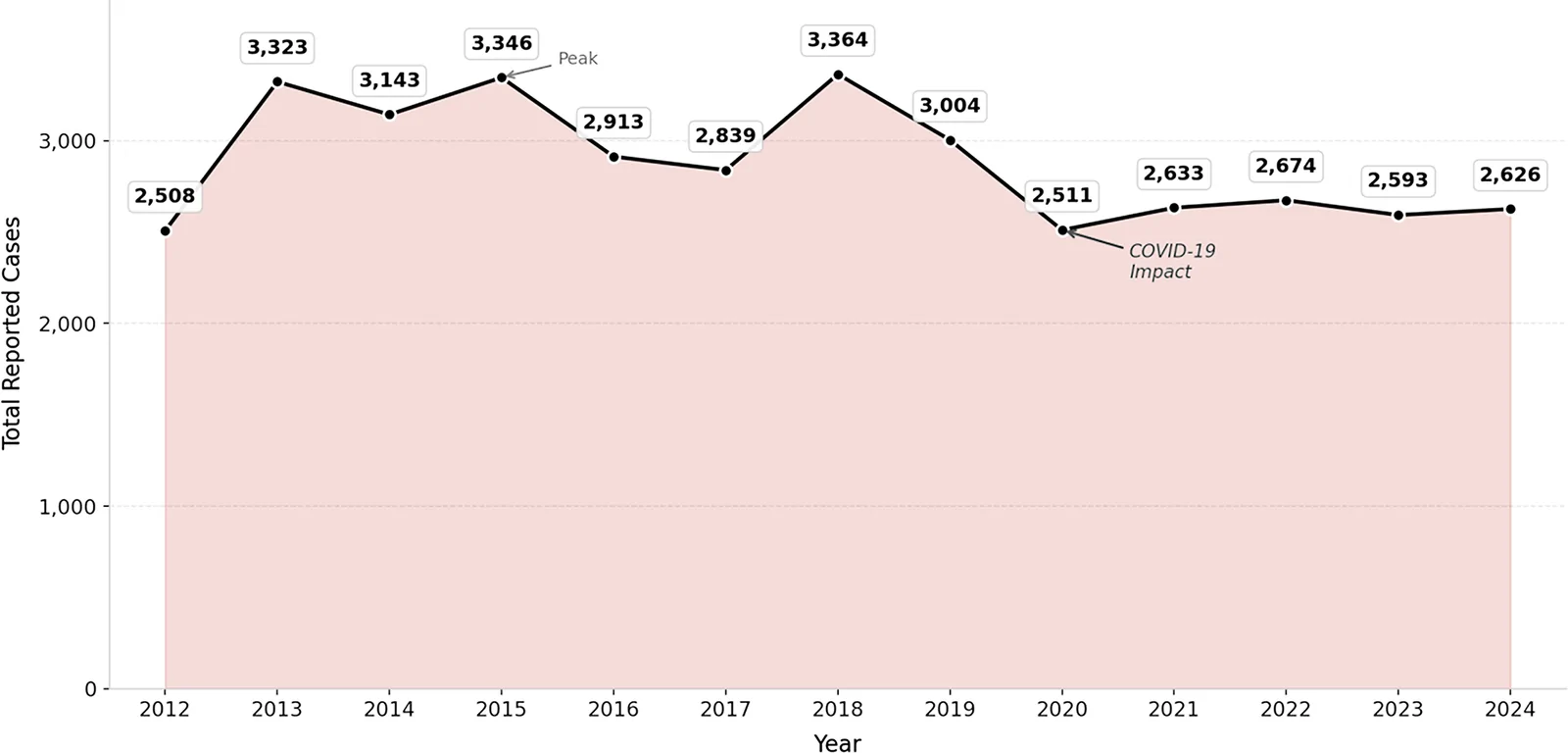
Tuberculosis case trends in Saudi Arabia (2012–2024)

Figure 2 shows that there were fluctuations over the first few years, followed by a decline in 2020, which was later followed by stabilization. For the period between 2021 and 2024, the number of cases reported annually was relatively constant, fluctuating between 2,593 and 2,674 cases.

The incidence rate of TB at the national level showed a similar trend; from 12.03 cases per 100,000 population in 2013 to 7.44 cases per 100,000 population in 2024 (Supplementary Table S1). Overall, PTB cases dominated among the total number of cases (73.6–79.0%), while EPTB comprised a stable proportion (21.0-26.4%) (Supplementary Figure S1).

**Table 1:** National tuberculosis burden and proportional distribution of pulmonary tuberculosis (PTB) and extrapulmonary tuberculosis (EPTB) in Saudi Arabia, 2012–2024

Year	Total Cases	Incidence (/100,000)	PTB *n* (%)	EPTB *n* (%)
2012	2,508	9.58	1,894 (75.5)	614 (24.5)
2013	3,323	12.03	2,447 (73.6)	876 (26.4)
2014	3,143	11.10	2,336 (74.3)	807 (25.7)
2015	3,346	11.22	2,505 (74.9)	841 (25.1)
2016	2,913	9.41	2,166 (74.4)	747 (25.6)
2017	2,839	9.16	2,095 (73.8)	744 (26.2)
2018	3,364	11.14	2,543 (75.6)	821 (24.4)
2019	3,004	9.99	2,264 (75.4)	740 (24.6)
2020	2,511	7.96	1,897 (75.5)	614 (24.5)
2021	2,633	8.55	1,970 (74.8)	663 (25.2)
2022	2,674	8.31	2,113 (79.0)	561 (21.0)
2023	2,593	7.69	2,017 (77.8)	576 (22.2)
2024	2,626	7.44	1,964 (74.8)	662 (25.2)

PTB = pulmonary tuberculosis; EPTB = extrapulmonary tuberculosis. Percentages indicate each TB type’s proportion of the annual national total

### Temporal trend and segmented regression analysis

**Fig. 3:**
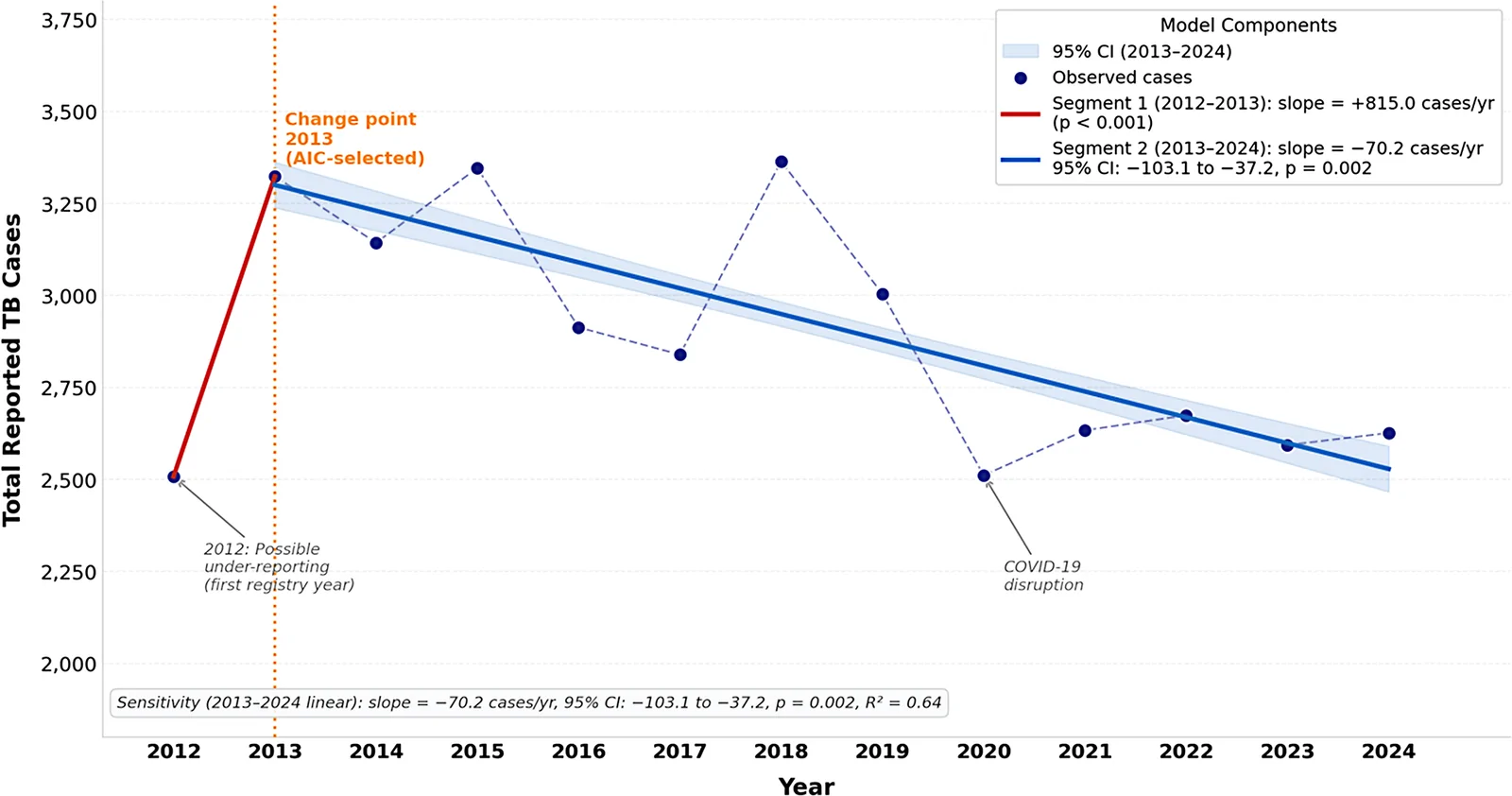
Segmented (piecewise linear) regression analysis for national tuberculosis notifications in Saudi Arabia (2012–2024)

The segmented regression analysis of TB case counts at the national level identified a single change point in 2013, which was chosen using Akaike Information Criterion (AIC) minimization among the possible breakpoints between 2013 and 2023. The 2013 breakpoint had the lowest AIC (144.5) among the candidate single-breakpoint models evaluated, which also included 2015 (AIC = 149.2), 2018 (AIC = 153.6) and 2020 (AIC = 154.7) (Fig. 3). Multi-breakpoint models were not formally fitted, and no claim of superiority over such alternatives is made. The three years, 2015, 2018 and 2020, show visible structural breaks, but none of these years yielded a lower AIC than 2013, and they are therefore treated as temporary deviations from a longer-term trend, instead of as structural breaks.

Notifications increased by 815 cases between 2012 and 2013. Because this interval contains only two annual observations, this value is reported as an observed one-year difference rather than as an estimated annual trend, and no *p* value is presented for this segment. This limitation arises when the identified change point falls at the boundary of the observation series. To our knowledge, 2012 also corresponds to an early operational year of the MOH digital TB surveillance registry, although an authoritative published source confirming this date could not be identified. The rise from 2,508 cases in 2012 to 3,323 cases in 2013 may therefore coincide with surveillance system expansion and progressive data capture maturation rather than a genuine epidemiological increase in TB transmission, although this cannot be verified from the available ecological surveillance data. Accordingly, no reliable trend inference is drawn from this initial segment.

Following the 2013 change point, a statistically significant and sustained decline was observed across the subsequent eleven years (2013–2024), with an estimated slope of − 70.2 cases per year (95% CI: −103.1 to − 37.2; *p* = 0.002; R² = 0.64), reflecting a consistent downward trajectory in TB notifications over the study period. Within this second segment, two notable transient deviations were observed: a secondary peak in 2018 (3,364 cases) and a pronounced trough in 2020 (2,511 cases), the latter temporally coinciding with COVID-19 pandemic disruptions to healthcare utilization, TB diagnostic services, and case reporting. Neither deviation, however, produced a lower AIC when evaluated as an alternative single breakpoint, which is consistent with the post-2013 declining trend remaining the dominant long-term pattern.

To assess the robustness of these findings in light of the potential surveillance artefact at the 2012 baseline, a pre-specified sensitivity analysis was conducted by restricting the model to the period 2013–2024 only. The resulting simple linear regression yielded an identical slope of − 70.2 cases per year (95% CI: −103.1 to − 37.2; *p* = 0.002; R² = 0.64), corroborating the primary model estimates. The convergence of primary and sensitivity model results supports the stability of the observed declining trend and reduces concerns regarding undue influence of the 2012 baseline on the overall conclusions.

### Tuberculosis distribution by nationality (2012–2024)

**Fig. 4:**
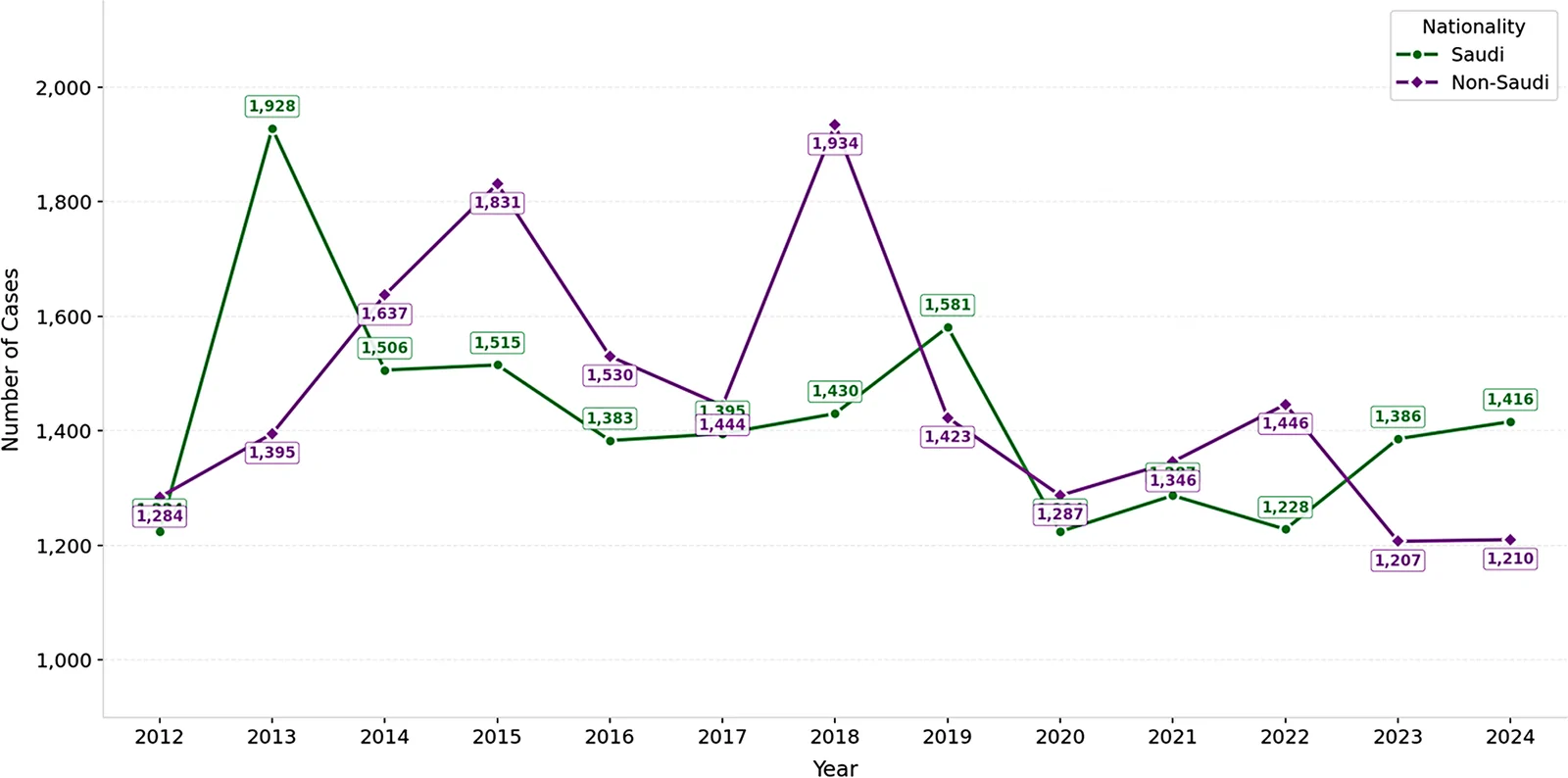
Cases of tuberculosis by nationality in Saudi Arabia (2012–2024). Absolute number of cases each year for Saudi citizens (green) and non-Saudi citizens (purple)

The ratio of TB cases between Saudi and non-Saudi nationals were temporally dynamic throughout the study period **(**Table 2; Fig. 4**).** Between 2012 and 2019, both nationalities accounted for relatively variable shares of the annual case burden, with Saudi nationals representing 42.5–58.0% and non-Saudi nationals representing 42.0-57.5%, respectively. Notably, 2018 recorded the highest proportion of non-Saudi cases in the pre-pandemic period (57.5%, *n* = 1,934), while 2013 recorded the highest Saudi proportion (58.0%, *n* = 1,928).

However, a shift occurred from 2020 onward, where non-Saudi nationals consistently exceeded Saudi nationals in annual case counts. This pattern was most pronounced in 2022, where non-Saudi nationals comprised 54.1% (*n* = 1,446) and Saudi nationals accounted for 45.9% (*n* = 1,228) of the total cases. As depicted in Fig. 4, the distribution showed a reversal in 2023 and 2024, where Saudi nationals comprised the majority at 53.5% and 53.9%, respectively (Supplementary Figure S2).

**Table 2:** Tuberculosis cases by nationality in Saudi Arabia (2012–2024)

Year	Saudi Nationals *n* (%)	Non-Saudi Nationals *n* (%)
2012	1,224 (48.8)	1,284 (51.2)
2013	1,928 (58.0)	1,395 (42.0)
2014	1,506 (47.9)	1,637 (52.1)
2015	1,515 (45.3)	1,831 (54.7)
2016	1,383 (47.5)	1,530 (52.5)
2017	1,395 (49.1)	1,444 (50.9)
2018	1,430 (42.5)	1,934 (57.5)
2019	1,581 (52.6)	1,423 (47.4)
2020	1,224 (48.7)	1,287 (51.3)
2021	1,287 (48.9)	1,346 (51.1)
2022	1,228 (45.9)	1,446 (54.1)
2023	1,386 (53.5)	1,207 (46.5)
2024	1,416 (53.9)	1,210 (46.1)

### Sex distribution of tuberculosis cases (2012–2024)

**Fig. 5:**
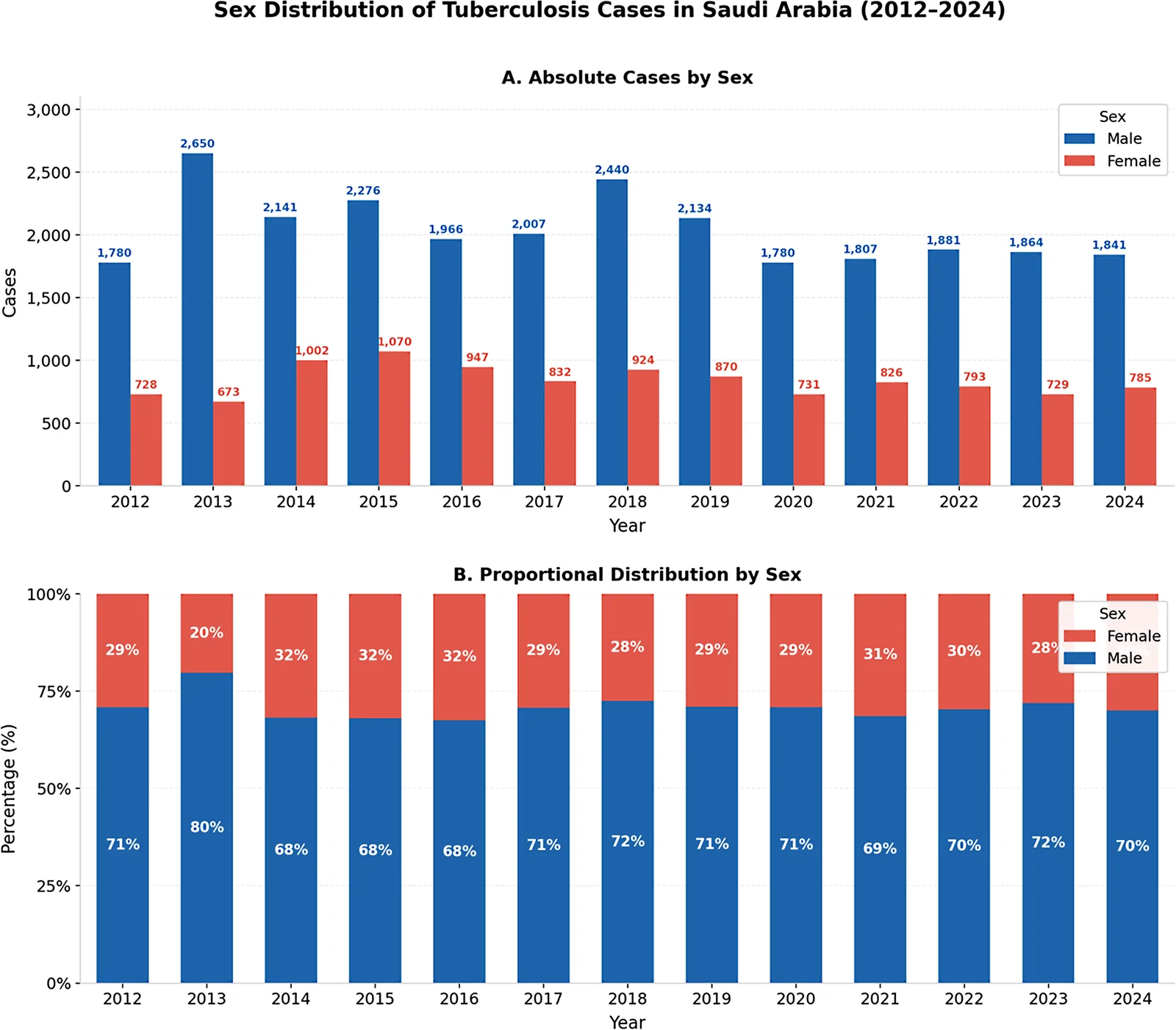
Sex-based distribution of TB cases in Saudi Arabia (2012–2024). **A**: Bar graph displaying absolute number of cases per year among males (blue bars) and females (red or salmon-colored bars). **B**: Distribution according to sex

Male dominance in TB cases was seen consistently throughout the study period **(**Table 3; Fig. 5**)**. Males constituted 67.5–79.7% of all TB cases reported, with the highest percentage recorded in 2013 at 79.7% (*n* = 2,650). The lowest male proportion was recorded in 2016 at 67.5% (*n* = 1,966), followed by 2015 at 68.0% (*n* = 2,276) and 2021 at 68.6% (*n* = 1,807). Female cases consistently comprised 20.3–32.5% of the annual total across all years.

In 2022, males accounted for 70.3% (*n* = 1,881) and females for 29.7% (*n* = 793), consistent with the overall pattern observed across the study period. The male-to-female ratio ranged from approximately 2.1:1 to 3.9:1 across the 13-year period.

**Table 3:** Sex distribution of tuberculosis cases in Saudi Arabia (2012–2024)

Year	Male *n* (%)	Female *n* (%)
2012	1,780 (71.0)	728 (29.0)
2013	2,650 (79.7)	673 (20.3)
2014	2,141 (68.1)	1,002 (31.9)
2015	2,276 (68.0)	1,070 (32.0)
2016	1,966 (67.5)	947 (32.5)
2017	2,007 (70.7)	832 (29.3)
2018	2,440 (72.5)	924 (27.5)
2019	2,134 (71.0)	870 (29.0)
2020	1,780 (70.9)	731 (29.1)
2021	1,807 (68.6)	826 (31.4)
2022	1,881 (70.3)	793 (29.7)
2023	1,864 (71.9)	729 (28.1)
2024	1,841 (70.1)	785 (29.9)

### Age group distribution of tuberculosis cases (2012–2024)

Throughout the study period, TB cases were most concentrated among working-age adults (Figs. 6a and 6b). The 25–34 years age group consistently recorded the highest annual case counts, peaking at 1,070 cases in 2013 and accumulating a total of 11,412 cases over the 13-year period, the single largest contributor to the overall burden. The 35–44 years and 15–24 years groups were the second and third highest contributors, with cumulative totals of 7,027 and 6,457 cases, respectively. Individuals aged 45–54 years were moderately represented (cumulative: 4,715), while those aged 55–64 and ≥ 65 years were less represented (3,415 and 3,262 cases, respectively). Pediatric TB cases, those aged under 5 years (343 cumulative) and 5–14 years (846 cumulative), remained consistently low throughout the study period. Age-group information was available for all 37,477 notifications, with no cases of unspecified age.

As illustrated in Fig. 6b, the proportional distribution of TB cases across age groups remained relatively stable over time with no discernible secular shift. This pattern indicates that reported TB notifications in Saudi Arabia were consistently concentrated among working-age adults, with the 25–34 year age group remaining the most frequently reported group across all years.

**Fig. 6a:**
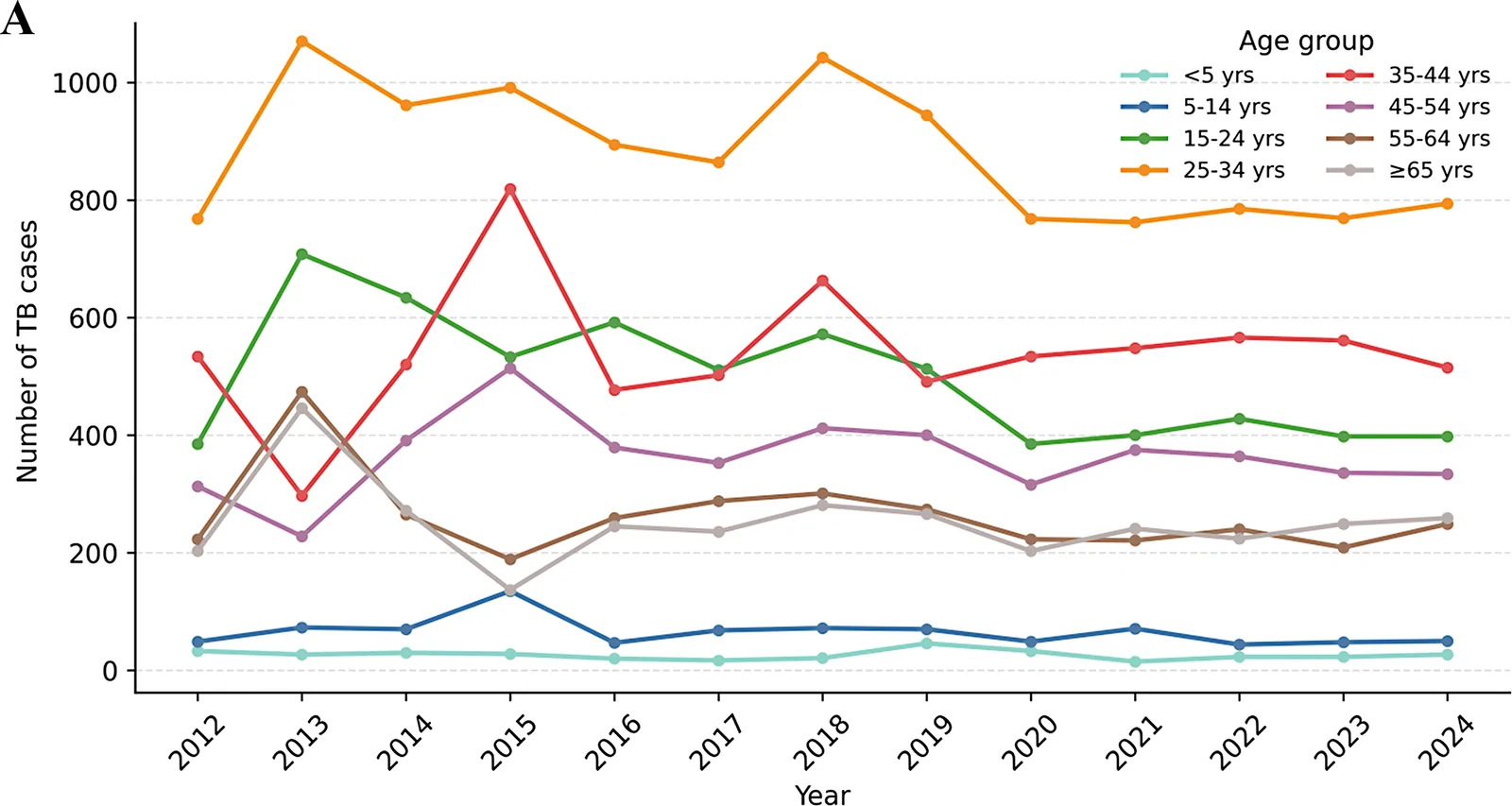
Trends in cases of tuberculosis by age groups in Saudi Arabia (2012–2024): absolute numbers. Multiline graph showing yearly numbers of TB cases (PTB + EPTB together) within eight age categories

**Fig. 6b:**
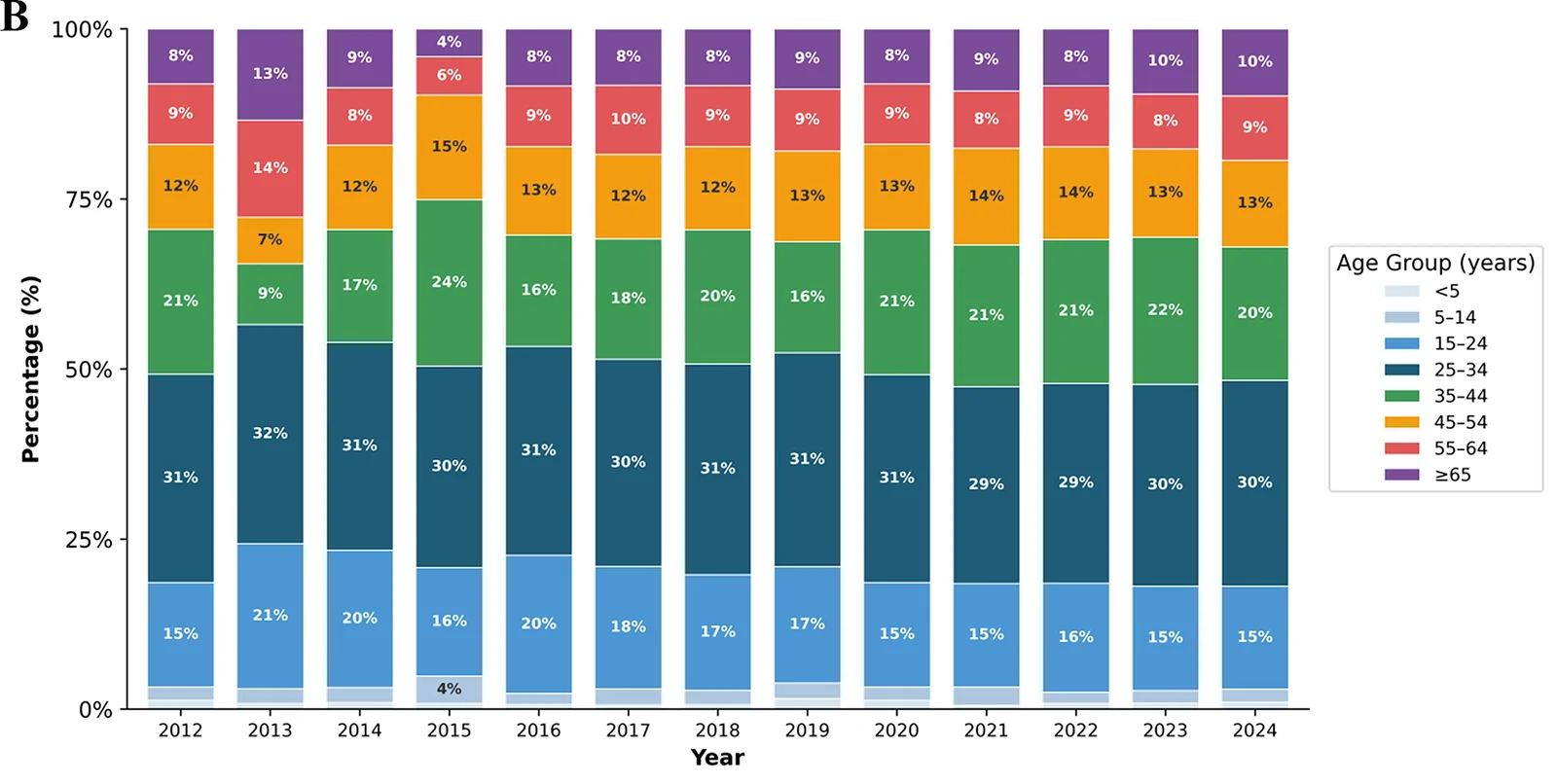
Age groups distribution of tuberculosis cases in Saudi Arabia (2012–2024). Distribution graph shows the annual proportionate contribution of each age group to national case counts of TB

### Regional distribution and spatial heterogeneity of tuberculosis (2012–2024)

**Fig. 7:**
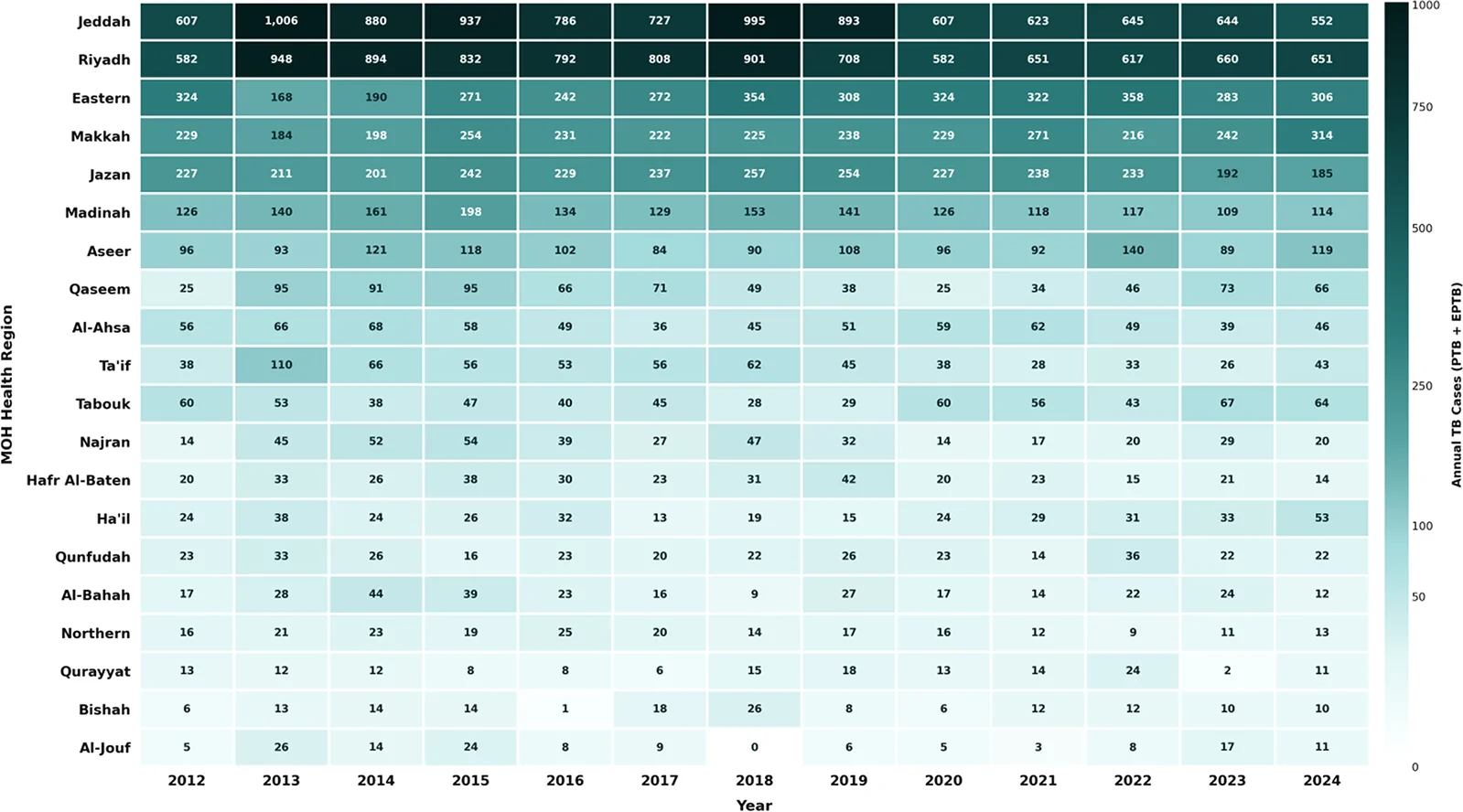
Regional heatmap for TB cases in Saudi Arabia (2012–2024). Heatmap depicting yearly total number of TB cases (PTB and EPTB together) by MOH health regions over the period. Each row shows a specific MOH health region ranked according to overall case burden, while each column shows a year in the calendar

**Fig. 8:**
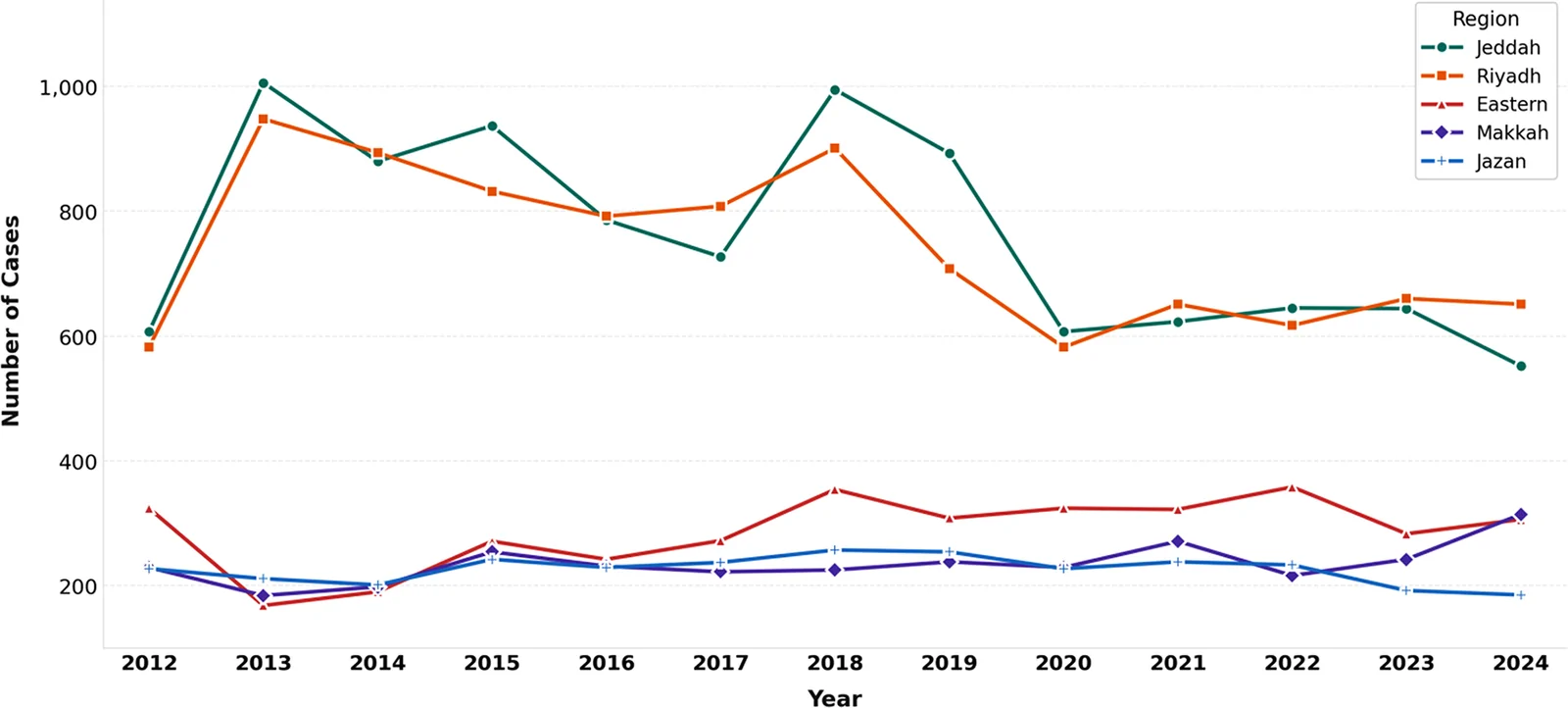
Top five tuberculosis case burden regions in Saudi Arabia (2012–2024). Line graph contrasting annual TB cases in five most represented Ministry of Health regions in Saudi Arabia: Jeddah (green), Riyadh (orange), Eastern (red), Makkah (purple), and Jazan (blue). Jeddah and Riyadh show a significant variation in annual figures

A considerable degree of geographical disparity in TB burden can be found within the 20 MOH health regions, as shown in Figs. 7 and 8, and Table 4.

The heat map in Fig. 7 highlights significant spatial variation in reported TB case counts over time. Jeddah and Riyadh recorded the highest annual case counts across all years under study, with annual figures ranging from 552 to 1,006 for Jeddah and 582 to 948 for Riyadh. High-intensity patterns were observed consistently throughout the study period, indicating a sustained concentration of cases in these two regions. Moderate case counts were recorded in the Eastern region, Makkah, and Jazan (168–358, 184–314, and 185–257 cases per year, respectively). Low case numbers were recorded in areas such as Al-Jouf, Bishah, Qurayyat, and Northern Borders, with fewer than 30 cases annually in most years, while Ha’il peaked at 53 cases in a single year.

The line chart depicted in Fig. 8 illustrates temporal trends across the five highest-burden regions. Jeddah and Riyadh demonstrate notable year-to-year variation, with pronounced peaks in 2013 (1,006 and 948 cases, respectively) and 2018 (995 and 901 cases), **a decline around** 2020, and subsequent stabilization. The Eastern region, Makkah, and Jazan show comparatively steadier trends with less pronounced fluctuations, ranging narrowly across years.

These findings are further supported quantitatively by Table 4. Over the 13-year study period, Jeddah contributed 9,902 cases (26.4%) of the national burden, while Riyadh ranked second with 9,626 cases (25.7%). The next highest-burden regions were Eastern (9.9%), Makkah (8.1%), and Jazan (7.8%). The remaining 15 regions collectively accounted for approximately 22.0% of total reported TB cases (Supplementary Figure S3).

While absolute case counts identify Jeddah and Riyadh as the highest-burden reporting regions, population-adjusted notification rates reveal a markedly different spatial pattern. Because official population denominators are published only for the 13 administrative regions of Saudi Arabia, and not for the 20 MOH reporting regions (several of which, such as Jeddah, Ta’if and Qunfudah, fall within the Makkah region), case counts were aggregated to the administrative-region level, where rates were calculated for the period 2012–2022. The cumulative burden analysis in Table 4, which requires no denominator, is retained at the 20-region level.

Jazan emerged as the region of greatest epidemiological concern, recording the highest rate in every disease category: a mean pulmonary TB (PTB) rate of 13.84 per 100,000 population (range 11.69–15.59), an extrapulmonary TB (EPTB) rate of 4.14, and a total-TB rate of 17.98. This is far higher than its modest 7.8% share of absolute cases would suggest. The Makkah region ranked second, at 11.11 (PTB), 3.18 (EPTB) and 14.29 (total TB), confirming that the concentration of cases around Jeddah reflects a genuinely elevated per-capita rate rather than population size alone. Riyadh, by contrast, recorded only moderate rates (6.71, 2.87 and 9.58, respectively) despite its large caseload, indicating that its burden is driven principally by its large population.

The remaining regions showed progressively lower rates, from intermediate total-TB values in Al-Bahah (8.03), the Eastern region (7.83) and Madinah (7.09) down to the lowest in Hail (3.77), Al-Jouf (4.32) and Qassim (4.65). Across all regions, EPTB contributed a smaller and more uniform share of the burden than PTB. Overall, the rate analysis reveals a spatial pattern that diverges meaningfully from the absolute-count distribution, with Jazan and the Makkah region carrying the highest population-adjusted burden and warranting priority in geographically targeted surveillance and active case-finding.

These results indicate that the TB burden in Saudi Arabia is geographically concentrated, but that per-capita incidence rates reveal a more complex spatial heterogeneity than absolute case distributions alone would suggest, with implications for equitable and targeted resource allocation across health regions.

**Table 4:** Cumulative tuberculosis burden (PTB + EPTB combined) by MOH health region in Saudi Arabia, 2012–2024

Health Region	Cumulative Cases (2012–2024)	% of National Total
Jeddah	9,902	26.4
Riyadh	9,626	25.7
Eastern	3,722	9.9
Makkah	3,053	8.1
Jazan	2,933	7.8
Madinah	1,766	4.7
Aseer	1,348	3.6
Qaseem	774	2.1
Al-Ahsa	684	1.8
Ta’if	654	1.7
Tabouk	630	1.7
Najran	410	1.1
Hafr Al-Baten	336	0.9
Ha’il	361	1.0
Remaining (6 regions)	1,278	3.4

### Regional trends of pulmonary and extrapulmonary tuberculosis

**Fig. 9:**
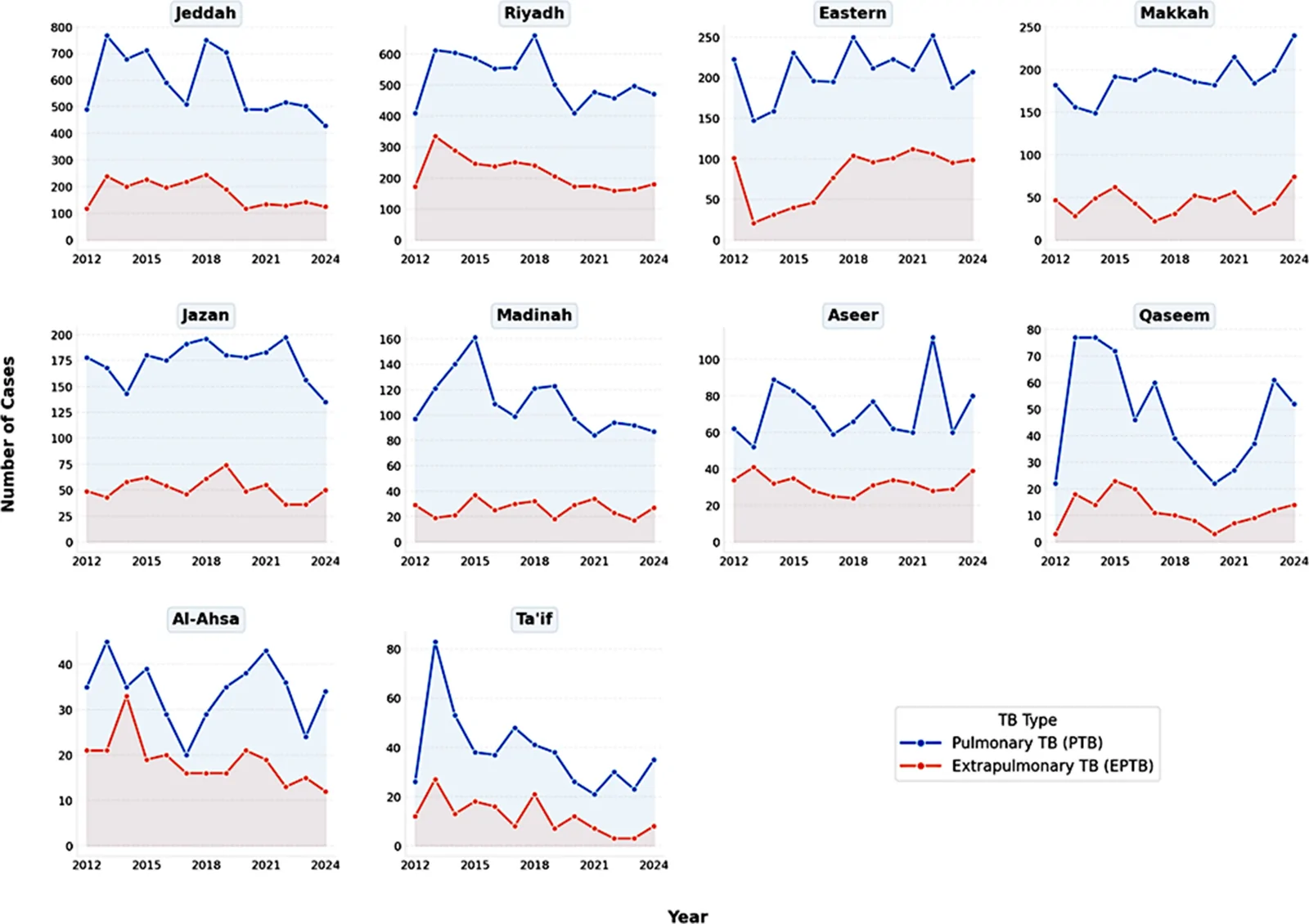
Trends in PTB and EPTB among the top ten health regions of the MOH in Saudi Arabia (2012–2024). Line graphs divided into two categories, showing annual numbers of cases of PTB (blue) and EPTB (red/salmon). The number of cases of PTB is always higher than that of EPTB in all regions

The faceted trend analysis plots of PTB and EPTB are presented for the top ten most represented health regions in Fig. 9 and Supplementary Figures S4 and S5 show the trend analysis for all health regions. It is evident from the panel-wise display that the cases were consistently more for PTB than EPTB throughout the observation period.

The number of cases of PTB ranged from 428 cases/year in Jeddah to 767 cases/year in Riyadh, and the number of cases of EPTB ranged from 117 cases/year in Jeddah to 335 cases/year in Riyadh. Case counts for Eastern region and Makkah were moderate with PTB at 147–252 and EPTB at 21–112, respectively, and at 149–240 and 22–74 respectively. The PTB count in Jazan ranged from 135 to 197 and EPTB was 36–74 per year.

Temporal trends were observed to be region-specific. For high burden cities like Jeddah and Riyadh, there were definite variations, with high levels around 2013 and 2018, and a decrease in 2020 before levelling off. Year-to-year variation was more pronounced in relation to the lower absolute numbers of cases in lower burden areas like Qaseem (PTB: 22–77; EPTB: 3–23), Ta’if (PTB: 21–83; EPTB: 3–27) and Al-Ahsa (PTB: 20–45; EPTB: 12–33). The trends of PTB and EPTB in each region followed broadly similar lines, but were not always in phase, implying that there are common but not identical epidemiological drivers for the two disease forms. None of the areas had a uniform pattern of change at all times. Complete PTB and EPTB trends for all 20 health regions are in Supplementary Figures S4 and S5.

### Forecasting of national tuberculosis incidence rates (2025–2029)

**Fig. 10:**
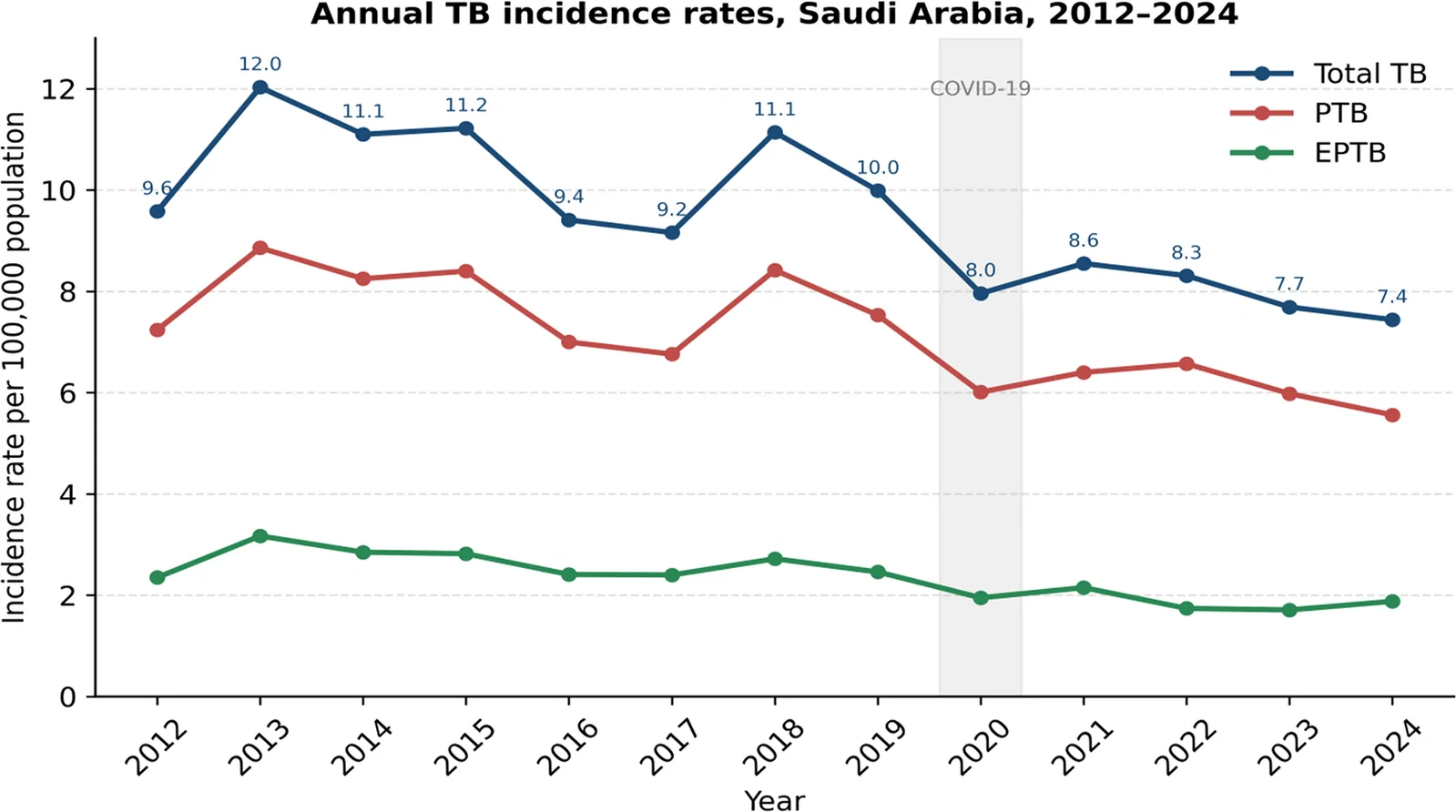
Annual incidence rates of pulmonary tuberculosis (PTB), extra pulmonary tuberculosis (EPTB), and total tuberculosis (TB) in Saudi Arabia, 2012–2024

Historical trends demonstrated a gradual decline in tuberculosis incidence rates across all disease categories between 2012 and 2024 (Fig. 10). PTB consistently exhibited higher incidence rates than EPTB throughout the study period and accounted for the majority of reported TB cases. Although annual fluctuations were observed, incidence rates generally declined after 2013, with a more pronounced reduction during the COVID-19 period.

**Fig. 11:**
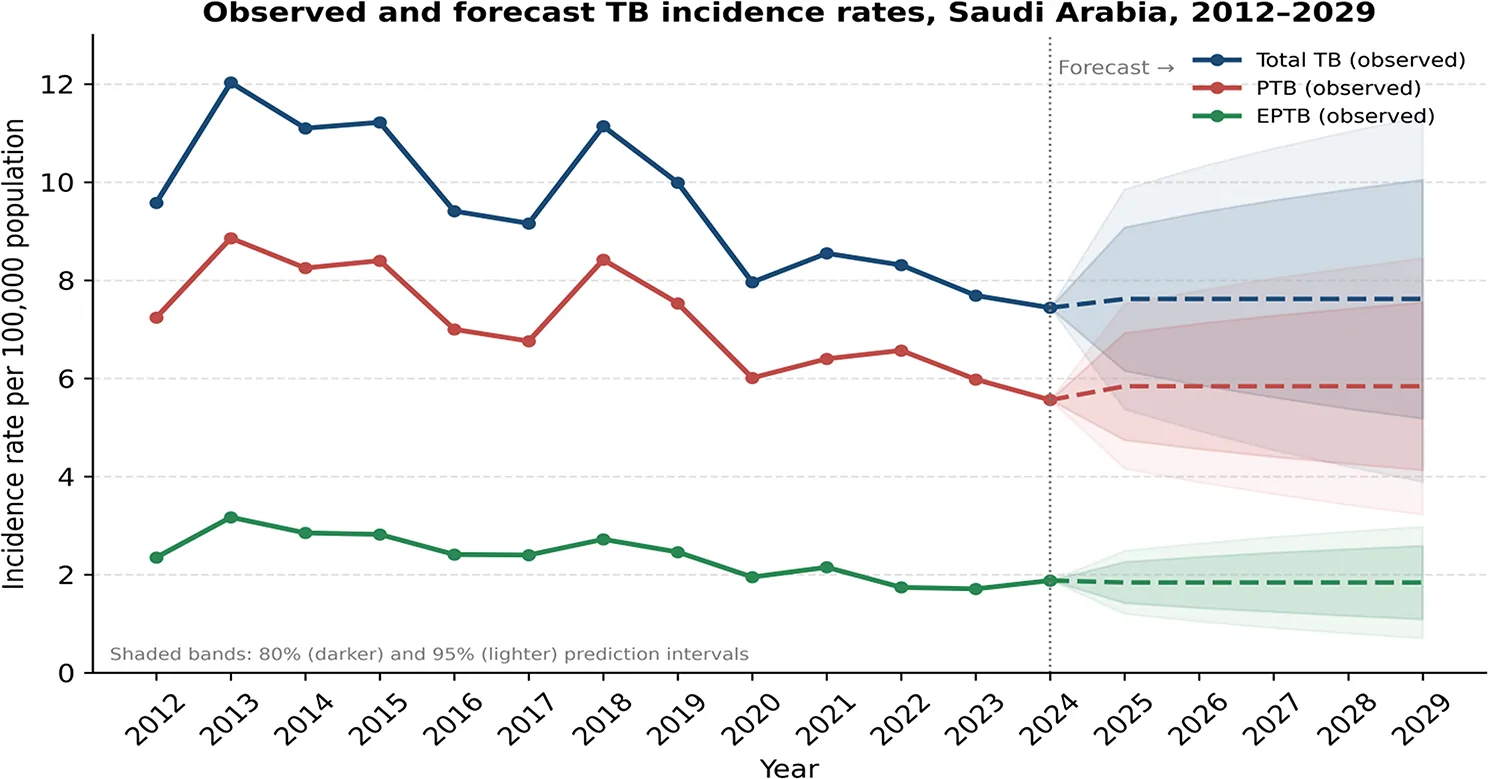
Forecasted incidence rates of pulmonary tuberculosis (PTB), extrapulmonary tuberculosis (EPTB), and total tuberculosis (TB) in Saudi Arabia, 2025–2029

ARIMA forecasting indicated a relatively stable epidemiological pattern during the projection period from 2025 to 2029 (Fig. 11). Forecasted PTB incidence remained close to 5.84 cases per 100,000 population, while EPTB incidence remained near 1.84 cases per 100,000 population throughout the forecast horizon. Consequently, total TB incidence was projected to remain around 7.62 cases per 100,000 population, suggesting minimal change from the levels observed in 2024. Because the models were fitted to only 13 annual observations, these forecasts should be regarded as exploratory and uncertain rather than as evidence that future incidence will remain stable. Full model specifications and annual forecasts with 80% and 95% prediction intervals are presented in Supplementary Table S2.

Prediction intervals widened progressively with increasing forecast horizons (Fig. 11), indicating greater uncertainty in long-term projections despite the stable central forecasts. Additional diagnostic assessments, including ACF/PACF analyses and residual diagnostics, indicated no substantial violations of model assumptions and supported model adequacy (Supplementary Figures S6–S8 and S12–S14).

Comparison with ETS models demonstrated a modest declining trajectory for all TB categories during 2025–2029 (Supplementary Figures S9–S11). PTB incidence decreased from 5.70 to 4.90 cases per 100,000 population, EPTB incidence from 1.69 to 1.33 cases per 100,000 population, and total TB incidence from 7.39 to 6.22 cases per 100,000 population over the forecast period. Overall, ARIMA and ETS forecasts indicated either stable or gradually declining incidence trajectories across all TB categories during the forecast period.

## Discussion

This study provides a comprehensive national-level assessment of epidemiology of TB in Saudi Arabia by integrating the epidemiological characteristics of TB over the past 13 years of TB surveillance data collected by the MOH, temporal trends, demographic stratification, geographical differences across all 20 health regions in Saudi Arabia and TB forecasting using ARIMA models. In general, there is evidence of a decreasing trend in TB notifications with some distinct spatial clustering. Notifications were concentrated among working-age adults (15–44 years) and were more frequently reported among males, while the proportion reported among non-Saudi residents varied over time and exceeded 50% in several years. The results also suggest that the number of cases has reached a post-pandemic plateau, highlighting the need for continued and intensified interventions to maintain and further reduce the number of cases.

The key insight from temporal trend analysis is the identification of 2013 as the statistical change point at which TB notifications transitioned from a rising phase to a sustained declining trend of − 70.2 cases per year (95% CI: −103.1 to − 37.2; *p* = 0.002), persisting through 2024. It should be noted, however, that the increase of 815 cases between 2012 and 2013 represents an observed one-year difference spanning only two annual observations, from which no reliable trend inference can be drawn. This initial change may coincide with surveillance system expansion and progressive data capture maturation rather than a genuine epidemiological surge, although this interpretation cannot be confirmed from the available data. The post-2013 declining trajectory aligns with global findings wherein the mid-2010s witnessed measurable improvements in TB control attributable to the adoption of standardized treatment protocols and enhanced case detection [26]. During this period, Saudi Arabia intensified pre-employment TB screening of expatriate workers and upgraded laboratory diagnostic capacities [9, 14]. The pronounced trough observed in 2020 (2,511 cases) temporally coincides with COVID-19 pandemic disruptions to health-seeking behavior, laboratory testing capacity, and case reporting, a pattern consistent with global evidence on pandemic-era TB service interruptions [27, 28]. Notably, post-pandemic stabilization between approximately 2,511 and 2,674 cases annually (2020–2024) did not recover to pre-pandemic peak levels, a pattern observed globally and potentially attributable to a combination of genuine incidence reduction and residual under-reporting effects [29, 30].

The shift in nationality distribution observed in 2022, wherein non-Saudi nationals comprised 54.1% of reported cases (*n* = 1,446) compared to 45.9% among Saudi nationals (*n* = 1,228), warrants careful contextualization. Critically, this represents a substantially more modest shift than previously suggested by uncorrected data and should not be interpreted as evidence of a dramatic epidemiological rupture. Rather, this modest elevation in non-Saudi proportions during 2021–2022, when non-Saudi nationals accounted for 51.1% (2021) and 54.1% (2022) of cases, likely reflects post-pandemic resumption of migrant labor inflows, comprising individuals from high-TB-burden countries in South Asia and sub-Saharan Africa, coinciding with intensified pre- and post-employment TB screening practices across Gulf states that may have augmented case detection without necessarily indicating altered transmission dynamics [31, 32]. Temporary disruptions in healthcare accessibility among Saudi citizens during the post-COVID recovery period may additionally have contributed to relative shifts in the proportional distribution by nationality. The return of nationality proportions towards pre-pandemic patterns in 2023 (Saudi 53.5%) and 2024 (Saudi 53.9%) supports the interpretation that the 2021–2022 deviation was transient rather than indicative of a structural epidemiological change [33]. These proportional patterns may be considered alongside existing literature reporting a high TB burden among populations originating from high-endemicity settings; because subgroup-specific population denominators were unavailable, the present data cannot establish differential risk by nationality [34–36].

The persistent male predominance in TB notifications, observed consistently across all years of the study, is well established in both global and regional literature [37]. Over the period 2012–2024, male cases constituted 67.5% to 79.7% of annual notifications, yielding male-to-female ratios ranging from approximately 2.1:1 to 3.9:1. Several factors have been proposed in the wider literature as possible contributors to this pattern, although none can be evaluated using the present ecological surveillance data. The expatriate workforce in Saudi Arabia is predominantly male, with a substantial proportion engaged in physically intensive occupations, including construction and agriculture, where overcrowded living and working conditions are recognized facilitators of TB transmission. Sociocultural and behavioral factors may additionally contribute, as men in many migrant-origin communities tend to delay healthcare-seeking, thereby prolonging infectious periods. Established biological and behavioral risk factors for TB, including tobacco use, are also more prevalent among men. Male predominance in TB burden is a globally recognized phenomenon acknowledged by the WHO in its annual global TB reports [7, 38, 39]. Notifications were also concentrated in the 15–44 years age group across all study years. Because sex, nationality, and age were not linked at the individual level and subgroup-specific denominators were unavailable, these distributions describe reported notifications and cannot demonstrate the contribution of any particular population group to national TB epidemiology [40].

Geographical analysis revealed a highly localized TB burden concentrated within a small number of densely populated urban health regions. Jeddah and Riyadh collectively accounted for 52.1% of the national cumulative burden (19,528 of 37,477 cases; 2012–2024), with Jeddah contributing 26.4% (9,902 cases) and Riyadh 25.7% (9,626 cases). This concentration likely reflects the demographic composition of these metropolitan centers, which host the largest concentrations of expatriate workers, as well as their comparatively superior case detection and diagnostic infrastructure [7, 41]. Moderate case burdens were recorded in the Eastern region (9.9%), Makkah (8.1%), and Jazan (7.8%), while the remaining 15 regions each contributed 4.7% or less of the national total. The relatively consistent case counts in Jazan and the Eastern region may reflect stable exposure patterns linked to local demographic compositions, whereas persistently low counts in regions such as Al-Jouf, Bishah, and Qurayyat (each recording fewer than 30 cases annually) are likely attributable to lower population densities and reduced non-Saudi concentrations. While these spatial patterns are suggestive of localized transmission dynamics, definitive inferences regarding intra-regional clustering require molecular epidemiological and contact tracing data not available in the current surveillance dataset. Nevertheless, these findings have direct implications for geographically targeted resource allocation and regional surveillance intensification [38, 42, 43].

The persistent predominance of pulmonary tuberculosis (PTB) over extra pulmonary tuberculosis (EPTB), ranging from 73.6% to 79.0% of annual cases across the study period, is consistent with surveillance trends reported in comparable epidemiological settings and is likely attributable to the greater clinical recognition and relative diagnostic ease of PTB compared with the more heterogeneous clinical presentations of EPTB [44–46]. The broadly synchronous temporal patterns of PTB and EPTB across high-burden regions suggest that the two disease forms are driven by shared epidemiological, demographic, and healthcare system factors, including exposure intensity and healthcare access, rather than disease-form-specific determinants [7, 47].

The final forecasting models were fitted to annual incidence rates per 100,000 population rather than to annual case counts. The central ARIMA forecasts remained close to 7.6 cases per 100,000 population through 2029. However, the wide and progressively expanding prediction intervals indicate substantial uncertainty regarding the future direction of TB incidence. Because the models were based on only 13 annual observations, these projections should be regarded as exploratory. Forecast precision decreases with increasing projection horizon, and the widening prediction intervals reflect increasing statistical uncertainty rather than anticipated epidemiological deterioration. These projections carry significant implications for TB control planning in Saudi Arabia. A plateau in notifications at current levels, without further decline, suggests that reliance on existing control strategies alone may be insufficient to meet the ambitious targets of the WHO End TB Strategy, which calls for a 90% reduction in TB incidence by 2030 relative to the 2015 baseline [48, 49]. Achieving more substantial reductions will likely require accelerated active case finding in expatriate communities with higher tuberculosis notification rates, systematic expansion of latent TB infection screening and treatment programs, and strengthened inter-ministerial coordination between health and labor regulatory bodies [50, 51]. The non-trivial upper prediction interval also serves as a reminder that higher burdens remain plausible under conditions of weakened surveillance capacity or disrupted control programs, as demonstrated by global experiences during the COVID-19 pandemic [52, 53].

Several limitations of this study should be acknowledged. First, the analyses were based on aggregated national surveillance data and are therefore subject to potential under-reporting, temporal changes in surveillance systems, reporting practices, case-detection activities, and regional variation in diagnostic capacity and surveillance completeness. In addition, individual-level information on HIV co-infection, treatment outcomes, drug resistance profiles, socioeconomic factors, and countries of origin of non-Saudi cases was unavailable. Second, subgroup-specific population denominators were not available for all demographic categories, and therefore sex-, nationality-, and age-specific findings should be interpreted as distributions of reported TB notifications rather than direct measures of relative risk or incidence. Third, regional incidence estimates relied on official MOH population denominators, which may not fully capture fluctuations in undocumented expatriate populations. Fourth, the absence of molecular epidemiological and transmission data precluded assessment of local transmission dynamics, clustering patterns, and importation pathways. Finally, forecasting analyses were based on only 13 annual observations and should therefore be regarded as exploratory, particularly for longer-term projections. Nevertheless, the use of a comprehensive 13-year national surveillance dataset, incidence-based regional analyses, and sensitivity analyses provides a useful epidemiological foundation for the study conclusions.

## Conclusions

The number of notified TB cases at national level decreased after the change point of 2013 and subsequently stabilized during the post-pandemic period. Although substantial progress has been achieved, the persistence of stable notification levels suggests that further reductions may be difficult to attain through existing control measures alone. Reported TB notifications remain concentrated in major urban health regions and were most frequently reported among working-age adults and males, with the proportion among non-Saudi residents varying over time and exceeding 50% in several years. Exploratory forecasting analyses suggest broadly stable incidence through 2029, although these projections are based on a small number of annual observations and should be interpreted cautiously. Strengthened surveillance, targeted case-finding and continued investment in prevention and control programs will be essential to support progress toward the WHO End TB Strategy targets. Future studies incorporating patient-level, molecular, and geospatial data may provide deeper insights into transmission dynamics and regional heterogeneity.

## Supplementary Information

Below is the link to the electronic supplementary material.

Supplementary Material 1

Supplementary Material 2

Supplementary Material 3

Supplementary Material 4

## Data Availability

All source data used in this study were obtained from publicly available Ministry of Health (MOH), Saudi Arabia, Statistical Yearbooks and Health Statistics reports for the years 2012-2024, available through the MOH statistical publications portal: https://www.moh.gov.sa/en/Ministry/Statistics/book/Pages/default.aspx. The study dataset was compiled from aggregated national surveillance tables contained within these reports. A cleaned and harmonized version of the dataset generated for the present study is available from the corresponding author upon reasonable request.

## Declarations

### Data transparency

All the data is available in main manuscript and its supplementary files.

## Abbreviations

TB: Tuberculosis
PTB: Pulmonary Tuberculosis
EPTB: Extrapulmonary Tuberculosis
PTB + EPTB: Combined Pulmonary and Extrapulmonary Tuberculosis Burden
MDR-TB: Multidrug-Resistant Tuberculosis
WHO: World Health Organization
MoH: Ministry of Health
GASTAT: General Authority for Statistics
ARIMA: Autoregressive Integrated Moving Average
ETS: Exponential Smoothing (Error, Trend, Seasonal)
ACF: Autocorrelation Function
PACF: Partial Autocorrelation Function
ADF: Augmented Dickey-Fuller (test)
KPSS: Kwiatkowski-Phillips-Schmidt-Shin (test)
AICc: Corrected Akaike Information Criterion
CI: Confidence Interval
PI: Prediction Interval
DOTS: Directly Observed Treatment, Short-course
R: R Statistical Software
RStudio: RStudio Integrated Development Environment
GIS: Geographic Information System
COVID-19: Coronavirus Disease 2019
HIV: Human Immunodeficiency Virus
