# Supplementary material for: Tuberculosis burden in Saudi Arabia: retrospective analysis of national surveillance data, 2012–2024, with future forecasts
Source: BMC Infect Dis. 2026 Aug 17;26:1995. doi: 10.1186/s12879-026-14222-5 (PMC13761192; doi:10.1186/s12879-026-14222-5)
Supplement: Supplementary file 1 [file 12879_2026_14222_MOESM4_ESM.docx]

**Tuberculosis Burden in Saudi Arabia: Retrospective Analysis of National Surveillance Data, 2012-2024, with Future Forecasts**

***Supplementary Figure S1.* The proportional distribution of pulmonary tuberculosis (PTB) and extrapulmonary tuberculosis (EPTB) in Saudi Arabia from 2012 to 2024**

***Supplementary Figure S2*. Proportionate distribution of tuberculosis patients based on nationality in Saudi Arabia (2012-2024).**

***Supplementary Figure S3*. Tuberculosis Cases Cumulated per Region in Saudi Arabia (2012-2024).** Bar graph illustrating the cumulative number of TB cases for all 20 health regions administered by the MoH.

***Supplementary Figure S4.* Trends of pulmonary tuberculosis (PTB) by region for all 20 regions of Saudi Arabian MoH (2012-2024).**

***Supplementary Figure S5.* Trends in extrapulmonary tuberculosis (EPTB) cases by region in all 20 health regions in Saudi Arabia (2012-2024).**

**Supplementary Figure S6.** ACF and PACF plots of annual pulmonary tuberculosis (PTB) incidence rates in Saudi Arabia (2012–2024) used for ARIMA model identification.

**Supplementary Figure S7.** ACF and PACF plots of annual extrapulmonary tuberculosis (EPTB)incidence rates in Saudi Arabia (2012–2024) used for ARIMA model identification.

**Supplementary Figure S8.** ACF and PACF plots of annual tuberculosis (TB) incidence rates in Saudi Arabia (2012–2024) used for ARIMA model identification.

**Supplementary Figure S9.** ARIMA and ETS forecasts of pulmonary tuberculosis (PTB) incidence rates in Saudi Arabia during 2025-2029.

**Supplementary Figure S10.** ARIMA and ETS forecasts of extrapulmonary tuberculosis (EPTB) incidence rates in Saudi Arabia during 2025–2029.

**Supplementary Figure S11.** ARIMA and ETS forecasts of tuberculosis (TB) incidence rates in Saudi Arabia during 2025–2029

**Supplementary Figure S12.** Residual diagnostics of the ARIMA model for pulmonary tuberculosis (PTB) incidence rates in Saudi Arabia (2012–2024), showing residual distribution, normality assessment, and residual autocorrelation.

**Supplementary Figure S13.** Residual diagnostics of the ARIMA model for extrapulmonary tuberculosis (EPTB) incidence rates in Saudi Arabia (2012–2024), showing residual distribution, normality assessment, and residual autocorrelation.

**Supplementary Figure S14.** Residual diagnostics of the ARIMA model for tuberculosis (TB) incidence rates in Saudi Arabia (2012–2024), showing residual distribution, normality assessment, and residual autocorrelation.
